# Guidance for the prevention and treatment of the post-thrombotic syndrome

**DOI:** 10.1007/s11239-015-1312-5

**Published:** 2016-01-16

**Authors:** Susan R. Kahn, Jean-Philippe Galanaud, Suresh Vedantham, Jeffrey S. Ginsberg

**Affiliations:** McGill University, Montreal, QC Canada; Department of Internal Medicine, Montpellier University Hospital, Montpellier, France; Mallinckrodt Institute of Radiology, Washington University School of Medicine, St Louis, MD USA; Department of Medicine, McMaster University, Hamilton, ON Canada; Division of Internal Medicine & Center for Clinical Epidemiology, Jewish General Hospital, 3755 Cote Ste. Catherine Room H420.1, Montreal, QC H3T 1E2 Canada

**Keywords:** Post-thrombotic syndrome, Venous thromboembolism, Deep venous thrombosis, Direct oral anticoagulants (DOAC), New oral anticoagulants (NOAC)

## Abstract

The post-thrombotic syndrome (PTS) is a frequent, potentially disabling complication of deep vein thrombosis (DVT) that reduces quality of life and is costly. Clinical manifestations include symptoms and signs such as leg pain and heaviness, edema, redness, telangiectasia, new varicose veins, hyperpigmentation, skin thickening and in severe cases, leg ulcers. The best way to prevent PTS is to prevent DVT with pharmacologic or mechanical thromboprophylaxis used in high risk patients and settings. In patients whose DVT is treated with a vitamin K antagonist, subtherapeutic INRs should be avoided. We do not suggest routine use of elastic compression stockings (ECS) after DVT to prevent PTS, but in patients with acute DVT-related leg swelling that is bothersome, a trial of ECS is reasonable. We suggest that selecting patients for catheter-directed thrombolytic techniques be done on a case-by-case basis, with a focus on patients with extensive thrombosis, recent symptoms onset, and low bleeding risk, who are seen at experienced hospital centers. For patients with established PTS, we suggest prescribing 20–30 mm Hg knee-length ECS to be worn daily. If ineffective, a stronger pressure stocking can be tried. We suggest that intermittent compression devices or pneumatic compression sleeve units be tried in patients with moderate-to-severe PTS whose symptoms are inadequately controlled with ECS alone. We suggest that a supervised exercise training program for 6 months or more is reasonable for PTS patients who can tolerate it. We suggest that management of post-thrombotic ulcers should involve a multidisciplinary approach. We briefly discuss upper extremity PTS and PTS in children.

## Introduction

The post-thrombotic syndrome (PTS) is a chronic condition that develops in ~20–50 % of patients after deep venous thrombosis (DVT) [1]. It adversely affects health and quality of life, and is costly as measured by health care costs, out of pocket expenditures, and lost productivity.

The objective of this chapter is to provide guidance for the general practitioner, internist, nurse practitioner, pharmacist, and other healthcare professionals on best current practices for the prevention and treatment of PTS.

## Background

Traditionally, clinical trials investigating new therapies or management approaches to treat DVT have focused on their effectiveness to prevent recurrent venous thromboembolism (VTE) in the short (3 months) to medium term (12 months) after DVT, while their effectiveness to prevent PTS has been ignored. Over the last 10–15 years, however, PTS has been increasingly recognized as a frequent and important outcome of DVT. Recent studies have improved understanding of the epidemiology, risk factors, and health and economic impact of PTS. Recommendations for standardization of the definition of PTS for clinical studies have been published [2], and rigorous clinical trials are underway to evaluate new approaches to preventing and treating PTS. Recently, the first evidence-based guidelines focused solely on PTS were published by the American Heart Association [3].

## Methods

To provide guidance on the management of the post-thrombotic syndrome, we first developed a number of pivotal practical questions pertaining to the PTS (Table 1). Questions were developed by consensus from the authors. The literature addressing the questions below was reviewed by searching electronic databases (PubMed, Medline) and the authors’ personal libraries, with a focus on high quality cohort studies and randomized controlled trials published in the last 10 years, where available. For each question, a brief summary and interpretation of pertinent literature and existing guidelines, where available, are provided, followed by guidance for the reader.

**Table 1:** Guidance questions to be considered

(1) What is PTS and why is it important (i.e. epidemiology, impact on quality of life, cost)?
(2) What are the clinical manifestations of PTS and what is its underlying pathophysiology?
(3) How is PTS diagnosed?
(4) What are known risk factors for PTS?
(5) Is there a best anticoagulant to treat DVT that influences the occurrence of PTS?
(6) What are current best approaches to preventing PTS after DVT?
(7) What are current best approaches to treating PTS?
(8) Does PTS occur after upper extremity DVT?
(9) Does PTS occur after DVT in children?
(10) What are the most pressing research needs in the field?

## Guidance

1. What is PTS and why is it important (i.e. epidemiology, impact on quality of life, cost)?

PTS is a clinical disorder of pain and disability resulting from chronic venous insufficiency following DVT. PTS is the most frequent complication of DVT. It develops in ~20–50 % of patients within 2 years of DVT diagnosis [4, 5], even when patients are adequately treated with anticoagulants, and is severe in 5–10 % of cases. Hence on average, about 6 of 10 DVT patients recover without any residual symptoms, 3 of 10 have some degree of PTS, and ~1 of 10 to 1 of 20 develop severe PTS that can include pain leg ulcers. The overall estimated incidence of VTE is 0.7–2 per 1000 person-years and increases with age [6, 7] so that more than one-third of cases occur in persons older than 60 years of age [8]. VTE is a growing public health problem due to increased life expectancy, an increasing proportion of elderly individuals and an expected increase in the prevalence of PTS.

Due to its high prevalence and chronicity, PTS is a costly condition. A Canadian study estimated that the total per-patient cost of PTS over a two-year period was almost 50 % higher than for DVT patients without PTS [9]. Costs were largely attributable to frequent healthcare visits and prescription medications. In the United States, annualized median total costs for DVT patients who developed PTS was $20,569 compared with $15,843 in matched controls with DVT and no PTS [10]. Costs are highest in those with PTS who develop venous ulcers, due to surgery, lost workdays and loss of employment [11]. It is estimated that 2 million workdays are lost annually in the United States due to leg ulcers [12].

Studies have shown that compared to DVT patients without PTS, patients with PTS have poorer quality of life [13–16] and scores worsen as severity of PTS increases [17]. Notably, PTS patients report worse quality of life scores than average scores for patients with osteoarthritis, diabetes and chronic lung disease [16].

### **Guidance Statement**

*Not applicable.*

(2) What are the clinical manifestations of PTS and what is its underlying pathophysiology?

The clinical manifestations of PTS are similar to those of primary venous insufficiency and include a constellation of symptoms and signs which vary from patient to patient [18] (Table 2). Typical symptoms include leg pain, a sensation of leg heaviness, pulling or fatigue, and leg swelling. Typical signs may include leg edema, redness, dusky cyanosis when the leg is in a dependent position, telangiectasia, new varicose veins, stasis hyperpigmentation, skin thickening and in severe cases, leg ulcers. The severity of symptoms and signs ranges from minimal discomfort and cosmetic concerns to severe clinical manifestations such as chronic pain, intractable edema, and leg ulceration [1, 19]. The intensity of symptoms and signs increases over the course of the day.

**Table 2:** Typical clinical features of the PTS

Leg symptoms	Signs
Heaviness or tiredness	Edema
Pain	Peri-malleolar telangiectasiae
Swelling	Venous ectasia, varicose veins
Itching	Hyperpigmentation
Cramps	Redness
Paresthesia	Dependent cyanosis
Bursting pain	Lipodermatosclerosis
Symptom pattern: worse with activity, standing, walking, better with rest, lying down, maximum at end of day	Healed ulcer(s) or open ulcer(s)

PTS is thought to develop after DVT due to venous hypertension (i.e. increased venous pressures) [20]. Venous hypertension leads to reduced calf muscle perfusion, increased tissue permeability and the associated clinical manifestations of PTS. Two pathological mechanisms contribute to venous hypertension: persistent (acute, then residual) venous obstruction (RVO) and valvular reflux caused by vein valve damage [21]. Inflammation may play a role in promoting the development of PTS by delaying thrombus resolution and by inducing vein wall fibrosis, which promotes valvular reflux [22, 23]. There may also be a genetic predisposition to PTS from gene polymorphisms associated mainly with vein wall remodelling [24].

### **Guidance Statement**

*Not applicable.*

(3) How is PTS diagnosed?

There is no gold standard laboratory, imaging, or functional test that establishes the diagnosis of PTS. PTS is primarily diagnosed on clinical grounds, based on the presence of typical symptoms and signs in a patient with previous DVT. Symptoms of PTS can be present in various combinations and may be persistent or intermittent. Symptoms tend to be aggravated by standing or walking and tend to improve with rest and leg elevation. In some patients, it can take a few months for the initial pain and swelling associated with acute DVT to resolve, thus a diagnosis of PTS should be deferred until after the acute phase (i.e. 3–6 months) has passed. Symptoms of PTS usually occur within 3–6 months after DVT, but can occur up to 2 years after DVT [25].

The Villalta PTS scale (sometimes called the Villalta-Prandoni scale) [26] has been adopted by the International Society on Thrombosis and Haemostasis (ISTH) as a standard to diagnose and grade the severity of PTS in clinical studies [2]. The scale’s components (5 symptoms and 6 signs) are each rated on a 4-point severity scale, and the points are summed to produce a total score; a score >4 denotes PTS (Table 3). The Villalta PTS scale has been shown to be valid, reproducible, and responsive to clinical change, and is easy to administer [27]. The Villalta PTS scale has been used to diagnose PTS in a number of recent randomized trials of interventions to prevent and treat PTS [28–32]. Additional diagnostic scales have been used to assess PTS, including the CAEP classification and Ginsberg measure; these are discussed in reference 2.

**Table 3:** Villalta PTS scale [2, 26]

Criteria used to diagnose PTS	
Assessment of	
5 symptoms (pain, cramps, heaviness, pruritus, paresthesia) by patient self-report	
6 signs (edema, skin induration, hyperpigmentation, venous ectasia, redness, pain during calf compression) by clinician assessment	
Severity of each symptom and sign is rated as 0 (absent), 1 (mild), 2 (moderate) or 3 (severe)	
Points are summed to yield total Villalta-PTS score	
0–4	No PTS
5–9	Mild PTS
10–14	Moderate PTS
15 or more, or presence of ulcer: severe PTS	

### **Guidance Statement**

*We suggest that in patients with a history of VTE, the Villalta PTS scale be used to assess the presence and severity of the PTS.*

(4) What are known risk factors for PTS?

While it is not yet possible to precisely predict the absolute risk of PTS in an individual patient with DVT, research done over the last 10 years has provided new information on various risk factors for PTS [1, 33]. This information is summarized below; the strongest risk factors are indicated with*:

*Risk factors apparent at time of DVT diagnosis*

- Age: Older age increases the risk of PTS.
- Elevated body mass index (BMI): Increased risk of PTS.
- Pre-existing primary venous insufficiency: Increased risk of PTS [34, 35].
- Characteristics of initial DVT: Risk of PTS is higher (2–3-fold) after proximal (especially with involvement of the iliac or common femoral vein) than distal (calf) DVT. Whether DVT was unprovoked vs. secondary (e.g. due to recent surgery, trauma, immobilization or active cancer) does not appear to influence the risk of developing PTS [5, 29].
- Gender, Inherited thrombophilia: No consistent relationship with PTS [33, 36].

*Risk factors related to treatment of acute DVT*

- Quality of oral anticoagulation: PTS risk increases (twofold) if level of anticoagulation is inadequate (e.g. subtherapeutic INR > 50 % time) during the first 3 months of treatment with vitamin K antagonists [37, 38].

*Risk factors apparent during follow*-*up after DVT*

*Recurrent ipsilateral DVT: Increases risk of PTS by 4–6-fold, presumably by damaging compromised venous valves or aggravating venous outflow obstruction [4, 5].
- Persistent venous symptoms/signs 1 month after acute DVT: Increased risk of subsequent PTS [5, 39].
- Residual thrombosis on ultrasound (e.g. 3–6 months after acute DVT): Modest (1.5–2-fold) increased risk of PTS [40].
- Persistent elevation of D-dimer: Elevated levels of D-dimer in the weeks to months after DVT may be a modest risk factor for PTS [41].

### **Guidance Statement**

*Not applicable.*

(5) Is there a best anticoagulant to treat DVT that influences the occurrence of PTS?

It is not known whether use of the new direct, target-specific oral anticoagulants to treat DVT influences the risk of PTS, compared to treatment with low molecular weight heparin (LMWH) and vitamin K antagonists [42]. Interestingly, some data suggest that use of LMWH monotherapy to treat DVT may lead to lower rates of PTS than standard treatment with LMWH followed by vitamin K antagonists [43]. These data require confirmation in large, well-designed RCTs.

### **Guidance Statement**

* Data are insufficient to make any recommendations regarding choice of anticoagulant, specifically a vitamin K antagonist vs. a target-specific oral anticoagulant, on the outcome of developing PTS.*

(6) What are current best approaches to preventing PTS after DVT?

*Primary prevention of DVT*

### **Guidance Statement**

* The best way to prevent PTS is to prevent the occurrence of DVT. We therefore suggest the use of pharmacologic or mechanical thromboprophylaxis to prevent VTE in high risk patients and settings, as recommended in evidence-based consensus guidelines* [44–46].

*Prevention of DVT recurrence*

As ipsilateral DVT recurrence is an important risk factor for PTS, preventing recurrent DVT by providing optimal anticoagulation of appropriate intensity and duration for the initial DVT is a key goal [47]. For specific suggestions on optimal anticoagulation to treat DVT, the reader is referred to the chapter in this volume titled ‘Guidance for the treatment of DVT and PE.

### **Guidance Statement**

*In patients whose DVT is treated with a vitamin K antagonist, frequent, regular INR monitoring should be performed to avoid subtherapeutic INRs, especially in the first 3 months of treatment.*

*Elastic compression stockings*

Elastic compression stockings (ECS), by reducing edema and venous hypertension, could plausibly play a role in preventing PTS. However, there are conflicting data on the long term effectiveness of ECS to prevent PTS. Two previous small, randomized, open label trials reported that wearing 30–40 mm Hg knee-high ECS for at least 2 years after proximal DVT was effective in preventing PTS [29, 48]. Based on these data, evidence-based consensus guidelines have recommended the use of ECS for at least 2 years after DVT to prevent PTS [47, 49]. However, a recent large (n = 803), multicenter, randomized, placebo-controlled trial showed no evidence of benefit of active compression stockings, worn for 2 years after proximal DVT, to prevent PTS: rates of PTS, recurrent VTE and QOL scores were similar in the active and placebo stockings groups [31]. Further, a secondary analysis of that trial showed no difference in pain scores during the first 60 days after DVT in the active and placebo stockings groups. The placebo-controlled blinded design of this trial is an important methodological strength, owing to the subjective nature of PTS assessment [50].

### **Guidance Statement**

* Based on these data, we do not suggest the routine use of ECS to prevent PTS in DVT patients, or to relieve acute DVT-related pain. However, because the trials cannot rule out a benefit of ECS in small sub-groups of patients or even to exploit a placebo benefit of ECS in patients with acute DVT-related leg swelling that is bothersome or uncomfortable, a trial of 20–30 mm Hg or 30–40 mm Hg ECS is not unreasonable.*

*Thrombolysis*

Upfront thrombolytic therapy in conjunction with heparin to treat acute DVT leads to higher rates of vein patency and better preservation of valve function than the use of heparin alone [21, 51]. Catheter-directed thrombolysis (CDT) or pharmacomechanical CDT (catheter-directed thrombolysis + mechanical disruption of thrombus) are likely to be safer and more effective than systemic thrombolytic therapy and could hold promise as a means of preventing PTS, primarily after proximal DVT [52]. In one multicenter randomized controlled trial of modest (n = 189) size, the use of additional CDT in anticoagulated patients with acute DVT involving the iliac and/or upper femoral vein was associated with a 26 % reduction in the risk of developing PTS over 2 years follow-up, with an additional 3 % rate of major bleeding [30]. Larger multicenter trials of PCDT+ standard anticoagulation vs. standard anticoagulation alone to prevent PTS are ongoing [53, 54] and results are expected within 1–2 years. The role of thrombolysis and other endovascular approaches in the management of DVT is discussed in greater depth in the Guidance for the use of Thrombolytic Therapy for DVT and PE chapter in this volume.

### **Guidance Statement**

*We suggest that selection of patients for catheter-directed thrombolytic techniques should be done on a case-by-case basis, with a predominant focus on patients with extensive (e.g. iliofemoral) thrombosis, recent onset of symptoms, low risk of bleeding and long life expectancy,* [47] *who are seen at hospital centers experienced in performing these techniques.*

(7) What are current best approaches to treating established PTS?

*Compression*-*based therapies*

A number of compression-based therapies have been used with the goals of reducing PTS symptoms (especially leg swelling and discomfort) and improving daily functioning. However, few controlled studies of their effectiveness have been performed, and available controlled studies are small, with limited follow-up time. Therefore, the suggestions below are based primarily on the low risk of harm and the possibility of benefit to at least some patients.

### **Guidance Statement**

*We suggest the following management approach for compression-based therapies: Prescribe 20–30 mm Hg ECS to patients with PTS-related leg heaviness or swelling. We suggest knee-length ECS, which have similar physiologic effects to thigh-length ECS and are easier to apply, more comfortable and less costly. Explain to the patient that these are to be worn daily, from waking to retiring. If 20–30 mm Hg ECS do not adequately control PTS symptoms, a stronger pressure stocking (30–40 mm Hg; or 40–50 mm Hg) can be tried* [32]. *We suggest that the portable, battery-powered Venowave*^®^*intermittent compression device be tried in patients with moderate to severe PTS whose symptoms are not adequately controlled with ECS alone* [28, 47]. *We suggest that intermittent pneumatic compression sleeve units (e.g. used for 20–30 min sessions, 2–3 times per day) can be used to help severe, intractable PTS symptoms or severe edema* [55], *however patients may find these to be cumbersome and the units are expensive.*

*Pharmacotherapy*

Four randomized trials have been performed to evaluate the effectiveness of “venoactive” drugs for PTS: three parallel trials [56–58] and one crossover study [59]. The drugs evaluated were rutosides (thought to reduce capillary filtration and microvascular permeability), defibrotide (down-regulates plasminogen activator inhibitor-1 release and up-regulates prostacyclin, prostaglandin E2, and thrombomodulin), and hidrosmin (mechanism of action unknown) [60]. Overall, there is low-quality evidence to support the use of venoactive drugs to treat PTS as studies were limited by a high degree of inconsistency and imprecision [60]. Also, as drug treatment was usually of short duration (e.g. 8 weeks to a few months), potential long-term side effects are unknown.

### **Guidance Statement**

*We do not suggest the use of venoactive drugs to treat PTS. Also, due to an absence of evidence and potential for harm, we do not suggest the use of diuretics to treat PTS-related edema*.

*Exercise and lifestyle*

Two small trials have assessed the effectiveness of exercise to treat PTS. In a study of 30 patients with chronic venous insufficiency (half had prior DVT), a six month leg muscle strengthening exercise program led to improved calf muscle function and calf muscle strength [61]. In a two-center Canadian pilot study, a 6 month program of exercise training that consisted of exercises to increase leg strength, leg flexibility and overall cardiovascular fitness improved PTS severity, quality of life, leg strength and leg flexibility, and there were no adverse events [62]. While not definitive, the available data suggest that exercise may benefit patients with PTS.

Common sense advice that is relevant to all patients with chronic venous insufficiency includes: promote venous return by avoiding a sedentary lifestyle, raising the legs on a stool when seated or elevating the legs in bed when lying down; avoid prolonged exposure to heat; maintain a healthy, non-obese body weight; and use a moisturizing cream to avoid skin dryness.

### **Guidance Statement**

*We suggest that a supervised exercise training program consisting of leg strengthening and aerobic activity for 6 months or more is reasonable for PTS patients who can tolerate it*.

*Venous ulcer management*

Five to 10 % of DVT patients develop severe PTS, which can include leg ulcers. Post-thrombotic venous ulcers are treated with compression therapy, leg elevation, topical dressings and sometimes hemorheological agents like pentoxifylline but can be refractory to therapy and tend to recur.

### **Guidance Statement**

* We suggest that ideally, management of patients with post-thrombotic ulcers involves a multidisciplinary approach that includes an internist, dermatologist, vascular surgeon and wound care nurse. For more detailed discussion of venous ulcer management, please refer to recent published reviews* [63, 64] *and consensus guidelines* [3].

*Surgical or endovascular treatments for PTS*

Surgical or endovascular procedures such as venous valve repair, venous bypass and venous stents to treat appropriately selected PTS patients have potential to decrease post-thrombotic manifestations that are attributable to deep vein obstruction or VR [65, 66]. However, because well-designed studies have not been performed to date, experience with these procedures varies substantially among providers, and rates of complications and failure are uncertain, these interventions should not be routinely utilized in unselected PTS populations. Rather, the opportunity to consult with an endovascular subspecialist who is experienced with the assessment and management of complex venous disease may be appropriate to discuss with selected patients with moderate-to-severe PTS who have substantial disability and life limitations. For more detailed discussion of surgical and endovascular treatments for PTS, please refer to a recently published AHA consensus guideline [3].

(8) Does PTS occur after upper extremity DVT?

After upper extremity DVT, 15–25 % of patients will develop PTS [67, 68]. As with lower limb PTS, upper extremity PTS can reduce quality of life and limb function [69, 70]. Not surprisingly, dominant arm PTS is associated with worse quality of life and disability than non-dominant arm PTS [69]. Data to guide the management of upper extremity PTS are lacking. There have been no trials of compression sleeves or bandages to prevent or treat upper extremity PTS, and it is not known whether thrombolysis, endovascular or surgical treatment of UEDVT results in lower rates of PTS than standard anticoagulation alone.

### **Guidance Statement**

*Due to potential for benefit and low potential for harm, we suggest a trial of a 20–30 mm Hg or 30–40 mm Hg compression sleeve in patients with symptoms of upper extremity PTS.*

(9) Does PTS occur after DVT in children?

The incidence of PTS is reported to be as high as 15–25 % in children with DVT [71, 72]. There are no pediatric studies that have evaluated the safety and effectiveness of therapies to prevent or treat PTS.

### **Guidance Statement**

* At present, we suggest that symptomatic management of PTS in children should generally follow adult guidelines, and that where possible, pediatricians with expertise in thromboembolism should manage pediatric patients with DVT.*

(10) What are the most pressing research needs in the field?

- Mechanistic studies to improve our understanding of the pathophysiology of PTS and to suggest future therapeutic targets
- Development of risk prediction indices to predict risk of PTS at the time of DVT diagnosis, in order to help guide the longitudinal management of patients with DVT
- Study of the role of risk factor modification (e.g. weight reduction, exercise) to prevent or treat PTS
- Assessment of the impact and cost-effectiveness of direct, target specific oral anticoagulants on the risk of PTS
- Assessment of the effectiveness, tolerability and cost-effectiveness of extended LMWH therapy to prevent PTS
- Studies of the effectiveness, safety and cost-effectiveness of PCDT to treat DVT as a means to prevent PTS
- Studies of the effectiveness of ECS and other compression modalities to treat lower extremity PTS, upper extremity PTS and pediatric PTS
- Assessment of the role of CDT/PCDT for prevention of upper extremity PTS and pediatric PTS
- Rigorous evaluation of the safety and long-term effectiveness of endovascular and surgical procedures to treat severe PTS

## Conclusion

PTS is a frequent complication of DVT that has the potential to reduce quality of life and lead to chronic functional disability. In this chapter, we have tried to provide guidance on key aspects relating to the diagnosis, risk factors, prevention and treatment of PTS (Table 4). Based on the numerous gaps in knowledge of PTS, we have also identified important areas for further research.

**Table 4:** Summary of guidance statements

Question	Guidance statement
(1) What is PTS and why is it important?	Not applicable; see text
(2) What are the clinical manifestations of PTS and what is its underlying pathophysiology?	Not applicable; see text
(3) How is PTS diagnosed?	We recommend that in patients with a history of VTE, the Villalta PTS scale be used to assess the presence and severity of the PTS
(4) What are known risk factors for PTS?	Not applicable; see text
(5) Is there a best anticoagulant to treat DVT that influences the occurrence of PTS?	Data are insufficient to make any recommendations regarding choice of anticoagulant, specifically, a vitamin K antagonist vs a target-specific oral anticoagulant, on the outcome of developing PTS
(6) What are current best approaches to preventing PTS after DVT?	For primary prevention
	Prevent the index DVT with use of thromboprophylaxis in high-risk patients and settings as recommended in evidence-based consensus guidelines
	For prevention of recurrent DVT
	In patients whose DVT is treated with a vitamin K antagonist, frequent, regular INR monitoring should be performed to avoid subtherapeutic INRs, especially in the first 3 months of treatment
	Value of elastic compression stockings
	We do not suggest the routine use of ECS to prevent PTS in DVT patients, or to relieve acute DVT-related pain. However, in patients with acute DVT-related leg swelling that is bothersome or uncomfortable, we suggest a trial of 20–30 mm Hg or 30–40 mm Hg ECS to relieve edema
	Value of thrombolysis
	The role of thrombolysis for the prevention of PTS is not yet established. In particular, pharmacomechanical catheter-directed thrombolysis requires further evaluation in properly designed trials. For now, we suggest that selection of patients for these techniques should be done on a case-by-case basis, and mainly considered for select patients with extensive thrombosis, recent onset symptoms, low bleeding risk and long life expectancy
(7) What are current best approaches to treating PTS?	Elastic compression stockings
	We suggest the use of 20–30 mm Hg (or stronger, if ineffective) ECS to reduce edema and improve PTS symptoms
	We suggest a trial of intermittent pneumatic compression devices in patients with moderate to severe symptomatic PTS
	Pharmacotherapy
	We do not suggest the use of venoactive drugs to treat PTS. Also, due an absence of evidence and potential for harm we do not suggest the use of diuretics to treat PTS-related edema.
	Exercise and lifestyle
	We suggest that a supervised exercise training program with leg strengthening and aerobic components for 6 or more months be tried in PTS patients who can tolerate it
	Management of venous ulcers
	We suggest a multidisciplinary approach to venous ulcer management, which usually consists of compression therapy, skin care and topical dressings
	In patients with symptoms of upper extremity PTS, we suggest a trial of a 20–30 mm Hg or 30–40 mm Hg compression sleeve
(8) Does PTS occur after upper extremity DVT?	Due to potential for benefit and low potential for harm, we suggest a trial of a 20–30 mm Hg or 30–40 mm Hg compression sleeve in patients with symptoms of upper extremity PTS
(9) Does PTS occur after DVT in children?	At present, we suggest that symptomatic management of PTS in children should generally follow adult guidelines, and that where possible, pediatricians with expertise in thromboembolism should manage pediatric patients with DVT
